# Strengthening continuity of care for multiple long-term conditions in India through integrated referral and follow-up systems (SYNC-RF): A mixed-methods study protocol

**DOI:** 10.1371/journal.pone.0361895

**Published:** 2026-11-03

**Authors:** Girish Chandra Dash, Samuel Seidu, Anneka Welford, Riya Patel, Patrick Highton, Kamlesh Khunti, Dorairaj Prabhakaran, Sailesh Mohan

**Affiliations:** 1 Diabetes Research Centre, University of Leicester, Leicester, United Kingdom; 2 Centre for Chronic Disease Control, Delhi, India; Universiti Sains Malaysia - Kampus Kesihatan, MALAYSIA

## Abstract

**Introduction:**

Multiple long-term conditions (MLTC) or multimorbidity are becoming increasingly common in low- and middle-income countries, such as India, placing demands on constrained primary healthcare systems that are still predominantly organised around acute care and single-condition management. Weak referral, counter-referral, and follow-up processes contribute to fragmented care and poor continuity for people living with MLTC. This programme of work aims to develop an integrated referral and follow-up model for people with MLTC who seek care in the primary healthcare system in India.

**Methods:**

This sequential, multistage mixed-methods research programme will be conducted in three interlinked phases. First, a systematic review of global literature will be conducted, which will synthesise evidence on referral and follow-up models or systems for providing MLTC care. Second, a convergent mixed-methods assessment will be undertaken in Jodhpur district, Rajasthan, including facility assessments of Primary Health Centres and linked referral facilities, a cross-sectional survey of adults with MLTC, and in-depth interviews with patients, caregivers, healthcare providers, and policymakers. Quantitative data will be analysed using descriptive and inferential statistics, while qualitative data will be analysed using reflexive thematic analysis. Third, an integrated referral and follow-up model, SYNC-RF (SYstem for Navigation, Coordination, Referral, and Follow-up), will be developed based on the triangulated evidence and refined through expert consultation to ensure feasibility and contextual relevance.

**Conclusion:**

The study will generate context-specific evidence on gaps, barriers, and enabling factors affecting referral and follow-up for MLTC care. The integrated model developed through this research is expected to strengthen continuity of care, improve coordination across various levels of the health system, and improve health outcomes for those living with MLTC.

## Introduction

Multiple long-term conditions (MLTC), also referred to as multimorbidity, are defined as the coexistence of two or more chronic conditions within an individual and represent a global public health challenge [1]. The prevalence of MLTC has increased substantially due to population ageing, epidemiological transition, and improved survival from chronic diseases, with recent global estimates suggesting that more than one-third of adults are affected [2]. Individuals living with MLTC often experience poorer health outcomes, including premature mortality, reduced quality of life, and increased healthcare utilisation, requiring coordinated and continuous care across multiple providers and levels of the health system, especially primary care [3].

In low- and middle-income countries (LMICs), such as India, the burden of MLTC is rising rapidly and increasingly impacting younger and middle-aged populations. Nationally representative data indicate that multimorbidity affects a significant proportion of adults, driven by the clustering of conditions such as hypertension, diabetes, chronic respiratory diseases, and cardiovascular disorders [4]. The prevalence of MLTC in India varies widely depending on population and case ascertainment methodology, with estimates ranging from 7.2% among younger adults to over 30–45% in older populations, and exceeding 50% in some community-based studies [4,5]. A recent systematic review further highlights substantial heterogeneity, reporting prevalence estimates ranging from 1.16% to 65.9% across studies in India, reflecting differences in study design, age groups, and disease definitions [6]. These conditions impose substantial financial and social burdens on individuals and households, particularly in a setting where out-of-pocket healthcare expenditure remains high [7].

Primary healthcare is widely recognised as the cornerstone of effective and equitable health systems and plays a central role in managing MLTC through prevention, early diagnosis, appropriate treatment, and long-term follow-up [8]. In India, Primary Health Centres (PHCs) serve as the first point of contact within the public health system and are expected to coordinate care through a three-tier referral structure linking PHCs with Community Health Centres (CHCs) and district hospitals. For individuals with MLTC, this referral continuum is essential, as they frequently require specialist consultations, diagnostic services, and ongoing monitoring across multiple levels of care [9].

Despite the importance of referral systems, evidence suggests that referral and follow-up mechanisms in many LMIC settings remain weak and poorly coordinated [10]. Studies have highlighted that referrals are often informal, inadequately documented, and lack standardised criteria, while counter-referral communication from higher-level facilities to primary care is frequently absent [11]. Inadequate follow-up systems further contribute to discontinuity of care, leading to missed appointments, duplication of services, delayed treatment, and poor patient outcomes [12]. These challenges are particularly pronounced for individuals with MLTC, who require integrated and longitudinal care pathways rather than episodic, disease-specific interventions.

Recent policy initiatives in India, including the expansion of non-communicable disease services under national programmes such as the National Programme for Prevention and Control of Non-Communicable Diseases (NP-NCD), National Programme for Prevention and Control of Deafness (NPPCD), National Programme for Control of Blindness (NPCB), and National Programme for Health Care of the Elderly (NPHCE), have strengthened screening and management at the primary care level [13]. However, these programmes largely remain disease-specific and provide limited operational guidance on managing patients with multiple coexisting conditions. Notably, there is a lack of structured frameworks to guide referral decision-making, information exchange across healthcare levels, and systematic follow-up for patients with complex health needs [14].

International evidence demonstrates that integrated referral pathways and care coordination models can improve continuity of care, patient outcomes, and health system efficiency [15]. However, most of these models have been developed in high-income settings and may not be directly applicable to resource-constrained, district-level health systems in LMICs [10]. Although existing studies have examined referral systems, care coordination, and continuity of care, important gaps remain in understanding how these mechanisms function for people living with MLTC within health systems in India. Much of the available evidence focuses on individual chronic conditions or specific components of healthcare delivery, while the referral and follow-up needs of people with multiple coexisting conditions require coordination across different services and levels of care. Furthermore, limited integrated evidence is available on how facility-level capacity, existing referral practices, patient and caregiver experiences, and healthcare providers’ perspectives collectively influence continuity of care for people living with MLTC. There is therefore a critical need for context-specific research to understand existing referral practices, identify system-level barriers and enablers, and develop integrated models tailored to primary healthcare settings in India.

This study addresses this important gap by generating empirical and contextual evidence on referral and follow-up systems for MLTC care, with the district serving as the unit for development of the integrated model within the Indian health system. By integrating global evidence with local health system assessment and stakeholder perspectives, the study aims to develop an integrated referral and follow-up model to strengthen continuity of care for people living with MLTC in primary healthcare.

The study is guided by three interrelated research questions corresponding to the three phases of the research programme: First, what referral and follow-up mechanisms have been reported for managing people living with multiple long-term conditions (MLTC), and what is known about their outcomes, implementation, and associated barriers and facilitators? Second, how are referral and follow-up systems for people living with MLTC organised and experienced within the selected district health system in India, and what patient-level and health-system factors are associated with referral completion and continuity of care? Third, how can evidence from the systematic review, facility assessment, patient survey, and stakeholder interviews be integrated to develop and refine a contextually appropriate referral and follow-up model for people living with MLTC within the existing Indian health system?

## Methods

### Study design

This study will use a sequential, multistage mixed-methods research design conducted in three interlinked phases [16]. Phase 1 will generate review-based evidence through a systematic review. Phase 2 will comprise a convergent mixed-methods assessment in which quantitative and qualitative data will be collected and analysed as complementary components to examine existing referral and follow-up systems within the district health system. Phase 3 will integrate findings from the preceding phases to develop and refine the System for Navigation, Coordination, Referral, and Follow-up (SYNC-RF) for people living with multiple long-term conditions (Fig 1). This design enables sequential progression across the overall programme while allowing convergence of quantitative and qualitative evidence within Phase 2. This design is intended to generate both empirical and contextual evidence to inform a feasible, patient-centred model for continuity of care for people living with multiple long-term conditions (MLTC).

**Fig 1:**
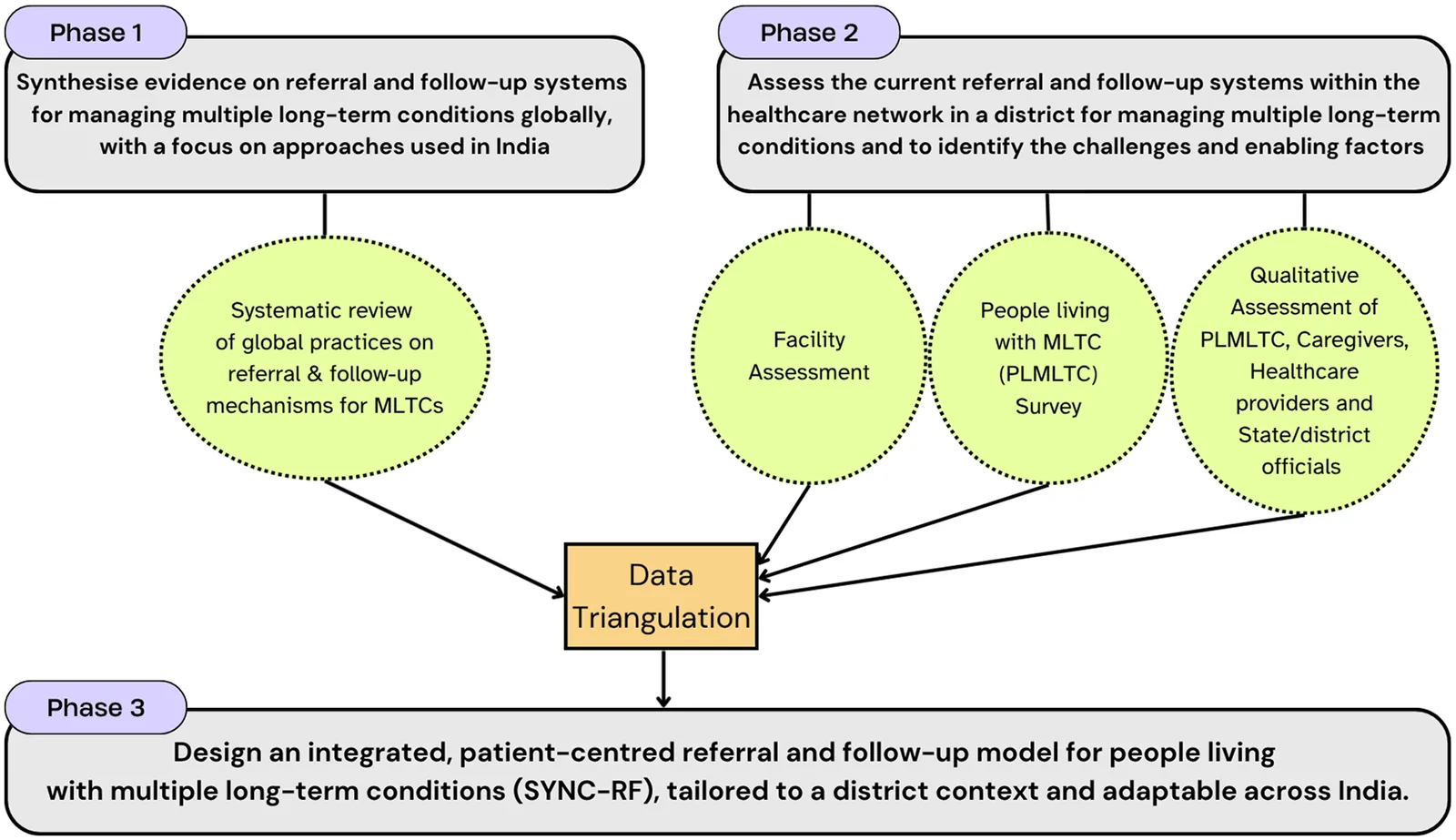
Development pathway for the integrated MLTC referral and follow-up model (SYNC-RF).

The three phases are interconnected through a structured process of evidence generation, contextual assessment, and model development. Phase 1 will synthesise existing evidence on referral and follow-up mechanisms for people living with MLTC, identifying relevant care coordination approaches, implementation strategies, and reported barriers and facilitators. These findings will provide an evidence base for interpreting the district-level assessment undertaken in Phase 2. The facility assessment will identify existing service capacity, referral pathways, documentation practices, and follow-up mechanisms, while the patient survey and qualitative interviews will examine referral completion, experiences of care, and perceived barriers and facilitators from different stakeholder perspectives. Quantitative and qualitative findings will be integrated to identify areas of convergence, complementarity, and divergence. In Phase 3, evidence from the systematic review and the integrated district-level assessment will be synthesised to identify the core components and operational requirements of the SYNC-RF model, including referral initiation, patient navigation, communication across levels of care, counter-referral, and follow-up tracking. Expert consultation will subsequently inform refinement of the model to enhance its contextual relevance, feasibility, and alignment with the existing Indian health system.

### Study setting

The empirical components of the study will be conducted in a district in India, where the district will be the operational unit for assessment and model development. In India, health is a state subject and within that the district is a key administrative and service-delivery unit through which care is organised across primary and secondary levels. It comprises a network of Sub-centres, Primary Health Centres (PHCs), linked Community Health Centres (CHCs), and a district hospital, which together form the referral continuum. PHCs serve as the first point of contact and are responsible for routine management, referral, and follow-up of patients with chronic conditions. Framing the district as the unit of analysis enables examination of referral and follow-up processes across interconnected levels of care and supports development of a model aligned with the structure of healthcare delivery in India.

### Phase 1: Evidence synthesis

#### Systematic review.

A systematic review will be undertaken to synthesise global evidence on referral and follow-up mechanisms for managing people living with MLTC. The review will be conducted in accordance with the Preferred Reporting Items for Systematic Reviews and Meta-Analyses (PRISMA 2020) guidelines [17], and the protocol title “Strengthening the Chain of Care: A systematic review of Referral and follow-up mechanisms for managing multiple long–term Conditions” has been registered with PROSPERO (Registration ID: CRD420251036215)

The eligibility criteria for the systematic review were developed using the SPIDER framework (Sample, Phenomenon of Interest, Design, Evaluation, and Research type), as this review aims to include quantitative, qualitative, and mixed-methods evidence on referral and follow-up systems for people living with multiple long-term conditions and are presented in Table 1.

**Table 1:** Eligibility criteria for the systematic review.

Element	Inclusion criteria
Sample	Adults aged 18 years and above living with multiple long-term conditions, defined as the coexistence of two or more chronic conditions
Phenomenon of Interest	Referral and/or follow-up mechanisms, including structured referral pathways, care coordination interventions, multidisciplinary models, patient navigation, counter-referral processes, electronic referral systems, and follow-up care models
Design	Quantitative, qualitative, and mixed-methods primary studies
Evaluation	Outcomes related to referral completion, follow-up adherence, continuity of care, patient or provider experiences, barriers and facilitators, implementation processes, and service delivery outcomes
Research type	Peer-reviewed quantitative, qualitative, and mixed-methods studies published in English; no date restrictions

This protocol has been checked against the PRISMA-P checklist (see S1 Appendix). The electronic databases to be searched will include MEDLINE (via PubMed), Embase, CINAHL, Scopus, Web of Science, and the Cochrane Library. Additional sources will include trial registries, reference list screening, and forward citation tracking. Search strategies will use a combination of controlled vocabulary and free-text terms related to multiple long-term conditions, multimorbidity, chronic disease, referral, follow-up, continuity of care, integrated care, and care coordination, and will be adapted for each database (see S2 Appendix).

Two reviewers will independently screen titles and abstracts, followed by full-text review of potentially relevant studies. Disagreements will be resolved through discussion, with involvement of a third reviewer where required. Data will be extracted independently by two reviewers using a pre-piloted standardised form capturing study characteristics, setting, population, intervention or model description, referral and follow-up components, outcomes, and implementation barriers and facilitators.

Risk of bias will be assessed using appropriate tools according to study design, including randomised studies, which will be appraised using the Revised Cochrane Risk of Bias Tool (RoB 2), cohort studies using the CASP Cohort checklist or another appropriate tool, other observational studies using a design-appropriate critical appraisal tool, and qualitative studies using the Critical Appraisal Skills Programme checklist. Findings will be synthesised narratively, with meta-analysis undertaken only if studies are sufficiently homogeneous in terms of design, intervention, and outcomes.

### Phase 2: Convergent mixed-methods assessment

#### Facility assessment.

A facility assessment will be conducted to evaluate the organisation, capacity, and performance of PHCs and their linked referral facilities regarding referral and follow-up systems for MLTC care. The assessment will cover selected PHCs, their linked CHCs, and the district hospital in the study district, thereby capturing the referral pathway across primary and secondary levels of care.

A total of 20 PHCs will be selected using stratified random sampling, comprising 10 rural and 10 urban PHCs. For each selected PHC, the corresponding linked CHC and the district hospital will also be included. This will allow assessment of the wider referral network and variation across different service settings.

Data will be collected using a structured facility checklist adapted from the Indian Public Health Standards and relevant national programme guidelines (see S3 Appendix) [18]. The checklist will cover service organisation, human resources, availability of essential medicines and diagnostics, referral documentation, communication systems, follow-up mechanisms, and programme-specific referral practices. Data sources will include facility records, registers, national programme reporting formats, staff inputs, and direct observation.

The analysis will be primarily descriptive. Indicators such as referral completion rate, counter-referral rate, turnaround time for referral, follow-up contact within 30 days, defaulter rate, and implementation status of relevant national programmes will be calculated. Comparative analysis will be undertaken across rural and urban PHCs and across levels of the referral system to identify strengths, gaps, and patterns in service delivery.

#### People living with MLTC Survey.

The primary objective of the survey will be to assess the frequency of referral to higher-level healthcare facilities or specialist services among people living with MLTC attending selected PHCs. Secondary objectives will include assessing referral completion, follow-up experiences, and patient-level and health-system factors associated with continuity of care. A cross-sectional survey will be conducted among adults aged 30 years and above attending the selected PHCs who have been clinically diagnosed with two or more chronic conditions. The conditions included in the study are diabetes mellitus, hypertension, oral, breast and cervical cancers, chronic obstructive pulmonary disease, asthma, chronic kidney disease, eye diseases, ear diseases, anaemia and non-alcoholic fatty liver disease (NAFLD), now called metabolic dysfunction-associated steatotic liver disease (MASLD). These conditions were selected because of their high prevalence, substantial contribution to morbidity and mortality, and major impact on healthcare utilisation in India. Several of these are included under the NP-NCD screening strategy at Primary Health Centres, while eye diseases, ear conditions, and anaemia were retained because of previously conducted formative work, which indicated that they are frequently encountered among PHC attendees.

Patients attending for acute conditions only and without a history of MLTC will be excluded. Individuals unable to provide informed consent will also be excluded unless accompanied by a caregiver who is permitted under the approved ethics protocol to provide proxy consent on their behalf. However, patients included through this provision must be able to participate in the survey and provide or confirm their own referral and follow-up information, with caregiver assistance where necessary. Caregivers often play an important role in managing healthcare and navigating referral pathways, particularly for older or dependent individuals. Where required, caregivers may assist patients in understanding survey questions, recalling referral and follow-up information, or communicating their responses. Their involvement will help ensure that the study captures referral and follow-up experiences that reflect real-world care practices. The source of survey responses will be recorded as either a direct patient response or a caregiver-assisted patient response. Each patient will remain the unit of analysis, and caregiver assistance will not constitute an additional observation. Caregivers will not provide independent survey responses on behalf of patients. Information that a patient is unable to provide or confirm, even with caregiver assistance, will be treated as missing rather than recorded as a caregiver-provided response.

The primary outcome of the patient survey will be the proportion of participants reporting receipt of a referral to a higher-level healthcare facility or specialist service. Referral completion within 30 days will be assessed as a secondary outcome among participants who received an eligible referral during the preceding 12 months. Referral initiation will be defined as a recommendation by a healthcare provider at a PHC for further assessment, investigation, or management at a higher-level healthcare facility or specialist service. Referral completion will be defined as actual attendance at the referred facility or another appropriate healthcare facility for the health concern that prompted the referral within 30 days of referral initiation. Scheduling an appointment without attending the referred service will not be considered referral completion. Referrals completed after 30 days will be classified as not completed within the primary outcome window but will be recorded separately as delayed completions. For urgent referrals requiring attendance within 24–72 hours, completion within the recommended timeframe will be assessed separately, alongside the primary 30-day outcome. Where participants report multiple referrals, the most recent referral occurring at least 30 days before the survey will be selected as the index referral for the primary patient-level analysis, while additional referrals will be summarised descriptively.

A total of 600 participants will be recruited, with a target of 30 participants from each of the 20 selected PHCs. The sample size was calculated to estimate the proportion of people living with MLTC who report receiving a referral to a higher-level healthcare facility or specialist service. A previous Indian study reported that 12.8% of patients attending a general outpatient department required specialist referral [19]. Using this estimate, a 95% confidence level and an absolute precision of ±3.5 percentage points, an initial sample size of approximately 350 participants was calculated using the single-proportion formula. To account for clustering of participants within PHCs, the sample size was adjusted using the design effect, DEFF = 1 + (m − 1) × ICC, where m represents the average cluster size and ICC the intracluster correlation coefficient. An ICC of 0.02 was assumed based on published primary-care research reporting a median ICC of 0.016 across study outcomes [20]. Given the fixed number of 20 PHCs, the cluster-adjusted sample size was calculated iteratively, yielding approximately 528 participants. Allowing 10% for incomplete data increased the required recruitment target to approximately 587 participants, which was rounded upwards to 600 to ensure equal allocation across the 20 PHCs. At each selected PHC, all adults attending the facility during the data-collection period will be screened for eligibility. Individuals meeting the study inclusion criteria will be approached consecutively and invited to participate. Those who provide informed consent will be enrolled until the target of 30 participants per PHC is reached.

Data will be collected using a pre-tested structured questionnaire administered by trained field investigators (see S4 Appendix). The questionnaire will include socio-demographic characteristics, MLTC profile, referral history, follow-up status, treatment burden, continuity of care, medication adherence, healthcare utilisation, out-of-pocket expenditure, and service use under relevant national programmes.

The analysis will include descriptive statistics to summarise participant characteristics, MLTC patterns, referral histories, and follow-up experiences. The proportion of participants reporting a referral to a higher-level healthcare facility or specialist service will be estimated with 95% confidence intervals, accounting for clustering within PHCs. Referral completion within 30 days will be assessed among participants who received an eligible referral. Exploratory analyses will examine factors associated with referral completion, including referral documentation and communication, age, sex, rural or urban residence, number of chronic conditions, socioeconomic status, and distance or travel time to the referred facility. Where the number of eligible observations and referral-completion outcomes permits, multivariable mixed-effects logistic regression with a PHC-level random intercept will be undertaken. The number of parameters included in the final model will be determined by the available outcome information to minimise unstable estimation. Adjusted odds ratios with 95% confidence intervals will be reported where appropriate, and the findings will be interpreted as exploratory associations rather than confirmatory hypothesis tests. The extent and pattern of missing data will be assessed before analysis. Where missing data are minimal, complete-case analysis will be undertaken; if missingness is substantial and considered compatible with a missing-at-random assumption, multiple imputation will be considered, with sensitivity analyses where appropriate.

#### Qualitative interview study.

A qualitative study will be conducted to examine how referral and follow-up processes shape continuity of care for people living with multiple long-term conditions within the district health system, in order to identify key barriers, facilitators, and system-level requirements to inform the proposed SYNC-RF model [21].

Participants will include (i) adults with MLTC who have experienced referral to a higher-level facility within the previous 12 months, (ii) caregivers involved in their care, (iii) healthcare providers working across Primary Health Centres, linked referral facilities, and district-level services, and (iv) district health officials involved in planning, implementation, or oversight of referral and follow-up systems. In-depth interviews (IDIs) will be used as the sole method of qualitative data collection. Purposive in-depth interviews will be conducted with 10 participants each from patients, caregivers, and healthcare providers, and with 4–6 state or district health officials [22]. The final sample size will be determined by information power and data saturation across various stakeholder groups.

Data will be collected using semi-structured in-depth interview guides informed by the study objectives and guided by the Chronic Care Model and the Consolidated Framework for Implementation Research (see S5 Appendix) [23, 24]. The use of these frameworks will support a structured exploration of continuity of care, referral pathways, follow-up experiences, communication across levels of care, and the barriers and facilitators influencing service use and implementation, while also informing development of the proposed model. Each interview is expected to last approximately 30–45 minutes and will be conducted in English and/or the local language, depending on participant preference. Interviews will be audio-recorded with consent and supplemented by field notes. Audio recordings will be transcribed verbatim and translated into English where necessary. Where interviews are conducted in the local language, transcripts will be translated into English by bilingual researchers familiar with the local context. Original-language transcripts will be retained alongside the translated versions, and culturally specific terms or expressions will be reviewed during analysis to minimise loss of meaning and contextual nuance.

Qualitative data will be analysed using reflexive thematic analysis [25]. Interview transcripts will first be read repeatedly to support familiarisation with the data. Initial coding will then be undertaken using a combination of deductive codes informed by the study objectives and inductive codes emerging from the interviews. Codes will be reviewed and grouped into sub-themes and broader themes. This will be developed iteratively to capture key experiences and system-level issues related to referral, follow-up, communication across levels of care, and barriers and enablers to continuity of care. Theme development and interpretation will be discussed within the research team to strengthen analytic rigor and ensure that findings remain grounded in participants’ accounts. Reporting of the qualitative findings will be guided by the Reflexive Thematic Analysis Reporting Guidelines (RTARG) [26].

#### Integration of quantitative and qualitative findings.

Quantitative and qualitative findings will be integrated through triangulation using a joint display matrix. Quantitative findings from the facility assessment and patient survey will be compared with qualitative themes from the in-depth interviews to identify areas of convergence, complementarity, and divergence across data sources. Where findings diverge, potential explanations will be explored by considering differences in stakeholder perspectives, referral practices, service settings, and available facility-level information. For example, discrepancies between patient-reported referral experiences and healthcare providers’ descriptions of referral practices may indicate gaps between intended service delivery and actual patient experiences. Such differences will not be automatically resolved in favour of one data source but will be retained as important findings requiring further interpretation. During Phase 3, both convergent and divergent findings will inform the identification of operational gaps and development of the SYNC-RF model. Unresolved discrepancies and their implications for model components will be presented during expert consultation to inform further refinement of the proposed model. This integrated interpretation will support a comprehensive understanding of how referral and follow-up systems function in practice, how they are experienced by patients and providers, and where key barriers and opportunities for strengthening continuity of care exist [27].

### Phase 3: Model development and expert consultation

Findings from the systematic review and mixed-methods assessment will be synthesised to develop an evidence-informed referral and follow-up model for MLTC care in primary healthcare. Model development will follow a structured approach informed by implementation and continuity-of-care perspectives and grounded in findings from the preceding phases, and will also include implementation frameworks, including the Chronic Care Model and the Consolidated Framework for Implementation Research Model.

Evidence from Phase 1 will be used to identify best practices, model components, and implementation lessons from global and Indian literature. Findings from Phase 2 will be used to define district-level strengths, gaps, and operational realities in referral and follow-up systems. These findings will be mapped across key domains such as referral initiation, documentation, communication pathways, patient navigation, counter-referral, follow-up tracking, and monitoring indicators. The model will be grounded in the existing Indian health system and informed by evidence from the review and current system assessment.

It will subsequently be refined through expert consultation. The panel will comprise approximately 8–12 experts, with 2–3 representatives from each of the four stakeholder groups: clinicians, public health specialists or health systems researchers, programme managers, and policymakers or senior health-system decision-makers, who will review the draft model for relevance, feasibility, clarity, and scalability using structured feedback and discussion-based consultation. Experts will be selected using criterion-based purposive sampling to ensure relevant expertise and approximately balanced representation. Potential experts will be approached individually and provided with information about the study and the draft SYNC-RF model. Those agreeing to participate will first be invited to independently review the model and provide structured feedback on its relevance, feasibility, clarity, acceptability, scalability, and alignment with the existing Indian health system. The individual feedback will then be collated and reviewed, and the draft model will be revised accordingly. A subsequent group consultation, conducted through an online or face-to-face meeting, will present the revised model to the experts and facilitate discussion of key modifications, areas of agreement, implementation considerations, and any remaining concerns. Final comments and recommendations from this consultation will be documented and used to refine and finalise the SYNC-RF model for potential adaptation and implementation in similar district health-system settings.

### Ethics and informed consent

Ethical approval was obtained from the Institutional Ethics Committee at the Centre for Chronic Disease Control, New Delhi (Ref No: IEC_09_2_2025, dated 03 October 2025), and the University of Leicester Ethics Committee (Ref No: 4820, dated 10 December 2025). Written informed consent will be obtained from all participants before enrolment. Where an individual is unable to provide informed consent, consent may be obtained from an accompanying caregiver acting as an appropriate proxy, where permitted under the approved ethics protocol. Proxy consent will not constitute permission for the caregiver to provide independent survey responses on behalf of the patient. Participation will be voluntary, and participants may withdraw at any time without affecting their healthcare.

### Study status and timeline

At the time of manuscript revision, the research programme is ongoing, with different components at varying stages of completion. The facility assessment was completed by mid-2026, while recruitment and data collection for the survey of people living with MLTC are ongoing and expected to be completed by the end of 2026. Qualitative data collection has commenced, with findings expected by the end of 2026. The systematic review is ongoing, with data extraction expected to be completed by the end of 2026. Expert consultation and refinement of the SYNC-RF model are expected to be completed by mid-2027. Final integration, analysis, and reporting of the programme findings are expected to be completed by the end of 2027.

## Discussion

The increasing burden of multiple long-term conditions (MLTC) presents a critical challenge for health systems globally, particularly in low- and middle-income countries where care delivery remains largely organised around acute care and single-disease models. This study protocol describes a mixed-methods approach to address gaps in referral and follow-up systems that undermine continuity of care for people living with multiple long-term conditions in primary healthcare settings. Rather than proposing a parallel structure, the study aims to develop the SYNC-RF model within the existing Indian public health system, using the district as the operational unit and building on current referral linkages between PHCs, CHCs, and district-level services. The study will therefore generate an evidence-informed model that is contextually relevant, feasible, and suitable for subsequent feasibility and acceptability testing before future evaluation in a randomised controlled trial. By integrating global evidence synthesis with a comprehensive assessment of district-level health system practices and patient experiences, this study adopts a systems-oriented approach to understanding referral and follow-up pathways. Previous research has demonstrated that fragmented care, weak communication between levels of care, and inadequate follow-up contribute significantly to poor health outcomes in patients with multimorbidity [28]. Notably, discontinuities in referral pathways have been associated with delays in diagnosis, treatment interruptions, and increased healthcare utilisation [29]. The present study builds on this evidence by examining both structural and experiential dimensions of care, enabling a more holistic understanding of how referral systems function in real-world primary healthcare settings.

The inclusion of facility assessments, patient surveys, and qualitative interviews allows for robust triangulation of data across multiple sources, thus strengthening the validity and applicability of findings. Similar mixed-methods approaches have been recommended in health systems research to capture complex interactions between service delivery, provider behaviour, and patient navigation [30]. By combining quantitative indicators—such as referral completion and follow-up adherence—with qualitative insights into barriers and enablers, this study is well positioned to generate actionable evidence for improving continuity of care.

A key contribution of this study lies in the development of an integrated referral and follow-up model tailored to the Indian primary healthcare context. Existing models of care coordination and integrated care for MLTC have largely been developed in high-income settings and may not adequately reflect the resource constraints and organisational realities of district-level health systems in LMICs. By grounding model development in empirical data and implementation frameworks such as the Chronic Care Model and the Consolidated Framework for Implementation Research, this study aims to enhance both the feasibility and scalability of the proposed intervention [31].

The study also has important policy implications. Strengthening referral and follow-up systems is essential for achieving continuity of care, improving patient outcomes, and optimising resource use within health systems [32]. In India, ongoing health system reforms emphasise comprehensive primary healthcare; however, operational guidance for managing MLTC remains limited. The findings from this study are expected to inform the design of context-specific, integrated care pathways that can be incorporated into existing national programmes and primary healthcare strengthening initiatives.

The study has some limitations. First, the empirical components are conducted within a single district, and variations in health-system organisation, infrastructure, resources, and referral practices across Indian states may limit the direct generalisability of the findings. However, the focus on common referral, communication, and follow-up processes within the district health system may provide transferable lessons for similar settings, while adaptation to local health-system contexts would be required before wider implementation. Second, the cross-sectional survey captures referral and follow-up experiences at a single point in time and therefore cannot establish causal relationships between participant or health-system characteristics and referral completion or follow-up outcomes. Third, as participants will be recruited from selected PHCs, the study may underrepresent people with MLTC who do not routinely access primary healthcare services. Although the inclusion of caregiver-assisted participation and proxy consent, where permitted under the approved ethics protocol, may facilitate participation of some older or dependent individuals, some vulnerable groups may still be underrepresented. Finally, translation of qualitative interviews into English may result in some loss of cultural nuance or locally specific meaning. This will be minimised by retaining the original-language transcripts and involving bilingual researchers familiar with the local context during translation and interpretation.

The focus on common health system processes such as referral coordination, information flow, and follow-up mechanisms shall allow for transferability of findings to similar settings across India and other LMICs. Future research would focus on piloting and evaluating the effectiveness of the proposed model in improving referral efficiency, continuity of care, and patient outcomes over time.

## Supporting information

S1 Appendix PRISMA-P checklist. (DOCX)

S2 Appendix Search strategy. (DOCX)

S3 Appendix Facility assessment checklist. (PDF)

S4 Appendix Survey questionnaire. (PDF)

S5 Appendix Interview guides. (PDF)

S6 Appendix Human participants research checklist. (DOCX)

S7 Checklist Inclusivity in global research questionnaire. (PDF)
