# Supplementary material for: Strengthening continuity of care for multiple long-term conditions in India through integrated referral and follow-up systems (SYNC-RF): A mixed-methods study protocol
Source: PLoS One. 2026 Nov 3;21(11):e0361895. doi: 10.1371/journal.pone.0361895 (PMC13730321; doi:10.1371/journal.pone.0361895)
Supplement: Supplementary file 1 [file pone.0361895.s003.pdf]

Facility Assessment Checklist
(Dash et al)

Section A – Facility Identification

1. Facility code:
2. Facility name:
3. Facility type: ☐ PHC ☐ CHC ☐ District Hospital ☐ Other (specify)
4. Location: ☐ Rural ☐ Urban
5. Address:
6. Name & designation of respondent(s):
7. Date of assessment:
8. Assessor name & designation:

Section B – Service Organisation and General Information

1. **Level of care provided:** ☐ Primary ☐ Secondary ☐ Tertiary
2. **Operating hours:**
    - OPD timings:
    - Emergency services: ☐ Yes ☐ No
3. Availability of inpatient services: ☐ Yes ☐ No (If yes, specify bed count)
4. Catchment population:
5. OPD attendance (day/week/month/year):
6. No of NCD cases or MLTCs in OPD (day/week/month/year):
7. Number of referrals received from lower facilities in last 6 months:
8. Number of referrals sent to higher facilities in last 6 months:

Section C – Human Resources

1. List of key staff positions (tick if available & currently filled):
    - Medical Officer(s) ☐ Yes ☐ No (Number:__)
    - Staff Nurse(s) ☐ Yes ☐ No (Number:__)
    - Pharmacist ☐ Yes ☐ No (Number:__)
    - Lab Technician ☐ Yes ☐ No (Number:__)
    - Physiotherapist ☐ Yes ☐ No (Number:__)
    - Community Health Officer ☐ Yes ☐ No (Number:__)
    - Counsellor (e.g., NCD counsellor) ☐ Yes ☐ No (Number:__)
    - Any Other Staff ☐ Yes ☐ No (Number:__)
2. Any vacant positions? List designation & duration vacant.
3. Availability of trained staff in:
    - NPCDCS protocols ☐ Yes ☐ No
    - NPHCE protocols ☐ Yes ☐ No
    - Referral documentation ☐ Yes ☐ No
    - Any other Protocol….. Mention

Section D – Infrastructure and Equipment

1. Availability of functional rooms/areas for:
   - OPD ☐ Yes ☐ No
   - NCD clinic ☐ Yes ☐ No
   - Laboratory ☐ Yes ☐ No
   - Pharmacy ☐ Yes ☐ No
   - Waiting area ☐ Yes ☐ No
   - Medical records section ☐ Yes ☐ No
2. Availability and functionality of essential equipment for chronic disease management:
   - BP apparatus (digital/manual) ☐ Yes ☐ No
   - Glucometer ☐ Yes ☐ No
   - ECG machine ☐ Yes ☐ No
   - Spirometer ☐ Yes ☐ No
   - Weighing scale ☐ Yes ☐ No
   - Height measuring scale ☐ Yes ☐ No
   - Stadiometer ☐ Yes ☐ No
   - Computer for data entry ☐ Yes ☐ No
3. Equipment maintenance log available? ☐ Yes ☐ No

Section E – Availability of Medicines & Diagnostics

1. **Essential medicines** for MLTCs (as per IPHS/NPCDCS list) available in last month:
   - Antihypertensives ☐ Yes ☐ No
   - Antidiabetics ☐ Yes ☐ No
   - Lipid-lowering agents ☐ Yes ☐ No
   - Analgesics ☐ Yes ☐ No
   - Inhalers/nebulisers ☐ Yes ☐ No
   - Others (list):
2. **Diagnostics available on-site**:
   - Blood glucose ☐ Yes ☐ No
   - Lipid profile ☐ Yes ☐ No
   - HbA1c ☐ Yes ☐ No
   - ECG ☐ Yes ☐ No
   - Urine protein ☐ Yes ☐ No
   - Other Tests Available (List)
3. If test not available, referral laboratory details:

Section F – Referral System ***(NHM indicators, MoHFW referral guidelines)***

1. Availability of written referral guidelines: ☐ Yes ☐ No
2. Referral documentation format available? ☐ Yes ☐ No
3. Referral registers maintained? ☐ Yes ☐ No

4. Referral slip includes:
    - Patient ID ☐ Yes ☐ No
    - Diagnosis ☐ Yes ☐ No
    - Reason for referral ☐ Yes ☐ No
    - Details of receiving facility ☐ Yes ☐ No
5. Do you perform or have any Referral tracking mechanism: ☐ Yes ☐ No
   If Yes,
    - % of referred patients confirmed to have reached higher facility (last 3 months)
6. Do you receive any information/feedback from the patients regarding their referral: ☐ Yes ☐ No
    - % of cases where feedback received (last 3 months)
7. Average turnaround time from referral to feedback:

Section G – Follow-up Mechanisms

1. Do you Follow-up after their visits? ☐ Yes ☐ No
   If Yes, Who does the followup – ASHA, ANM, Other Specify.
2. Standard follow-up schedule maintained? ☐ Yes ☐ No
3. Follow-up registers available? ☐ Yes ☐ No
4. Mechanism for contacting patients who miss follow-up:
    - Phone call ☐ Yes ☐ No
    - Home visit ☐ Yes ☐ No
    - Other (specify):
5. Follow-up within 30 days of referral completion: ☐ Yes ☐ No (% of patients)
   If not average no of days taken for referral completion:

Section H – Health Information Systems

1. Mode of record-keeping: ☐ Paper ☐ Digital ☐ Both
2. Use of HMIS/NCD portal for reporting: ☐ Yes ☐ No
3. Completeness of reporting in last quarter (%):
4. Data linkage with higher referral facilities: ☐ Yes ☐ No

Section I – Programme Implementation Status

1. NPCDCS clinic functioning: ☐ Yes ☐ No
2. NPHCE clinic functioning: ☐ Yes ☐ No
3. Other relevant programmes (list):
4. Availability of programme-specific registers: ☐ Yes ☐ No

Section J – Observations & Comments

- General remarks on facility readiness:
- Gaps identified:
- Good practices observed:
