# Supplementary material for: Strengthening continuity of care for multiple long-term conditions in India through integrated referral and follow-up systems (SYNC-RF): A mixed-methods study protocol
Source: PLoS One. 2026 Nov 3;21(11):e0361895. doi: 10.1371/journal.pone.0361895 (PMC13730321; doi:10.1371/journal.pone.0361895)
Supplement: Supplementary file 2 [file pone.0361895.s004.pdf]

People living with MLTC Survey questionnaire
(Dash et al)

Section A – Patient Identification & Demographics

1. **Study ID**: _______
2. **Date of Interview**: ___ / ___ / _____
3. **Interviewer ID**: _______
4. **Name**: _______
5. **Contact Number**: _______
6. **Address:** _______
7. **Age (in completed years)**: ______
8. **Sex**: ☐ Male ☐ Female ☐ Other
9. **Marital Status**: ☐ Single ☐ Married ☐ Widowed ☐ Divorced/Separated
10. **Education Level**:
    ☐ No formal schooling
    ☐ Primary
    ☐ Secondary
    ☐ Higher secondary
    ☐ Graduate & above
11. **Occupation**: ☐ Unemployed ☐ Self-employed ☐ Salaried ☐ Agricultural labourer ☐ Other __________
12. **Household Monthly Income (₹)**: ☐ <5,000 ☐ 5,000–10,000 ☐ 10,001–20,000 ☐ >20,000
13. **Socio-economic status (if applicable)**: BPL Card ☐ Yes ☐ No
14. **Residence**: ☐ Urban ☐ Rural
15. **Distance from facility**: _____ km

Section B – Health Conditions & Medical History

1. **Number of diagnosed long-term conditions**: ___

2. **List of conditions** (tick all that apply):
☐ Diabetes ☐ Hypertension ☐ Chronic respiratory disease ☐ Heart disease ☐ Chronic kidney disease ☐ Stroke ☐ Cancer ☐ Arthritis ☐ Other: ___________
3. **Year of first diagnosis for first condition**: ____
4. **Year of most recent diagnosis**: ____
5. **Where were you first diagnosed?**:
☐ PHC ☐ CHC ☐ District Hospital ☐ Private Clinic/Hospital ☐ Other ___________
6. **Current treatment facility**:
☐ Same as diagnosis facility ☐ Referred elsewhere (specify) _________
7. **Current medications** – list & duration: ________________________

Section C – Referral & Follow-up Experience

1. **Have you ever been referred to a higher-level facility for any of your conditions?**
☐ Yes ☐ No
2. If **Yes**:
    - **Who referred you?** ☐ Doctor ☐ Nurse ☐ Other ___________
    - **Where were you referred?** (facility type/name) ___________
    - **Reason for referral**: ☐ Diagnostic tests ☐ Specialist opinion ☐ Emergency care ☐ Procedure/surgery ☐ Other ___________
    - **Was referral documented?** ☐ Yes (referral slip) ☐ No
    - **Time taken to reach referred facility**: _____ hours/days
    - **Did you go to the referred facility?** ☐ Yes ☐ No – Reason if No: ☐ Cost ☐ Distance ☐ No transport ☐ Felt not needed ☐ Other ___________
3. **Have you received a follow-up call/visit from the referring facility?** ☐ Yes ☐ No
4. **Do you get regular follow-up appointments for your conditions?** ☐ Yes ☐ No
5. **Average time gap between follow-ups**: _____ weeks/months
6. **Do you receive counter-referral feedback from higher facilities?** ☐ Yes ☐ No
7. **Is you ABHA ID Created?** ☐ Yes ☐ No
8. **Are your health records shared between facilities?** ☐ Yes ☐ No

Section D – Service Utilisation

1. **Number of outpatient visits for your conditions in the last 3 months/12 months**: ____
2. **Number of hospital admissions in the last 12 months**: ____
3. **Were all prescribed medicines available at the facility?** ☐ Always ☐ Sometimes ☐ Never
4. **Were required diagnostic tests available at the facility?** ☐ Always ☐ Sometimes ☐ Never
5. **Have you missed follow-up visits in the last 12 months?** ☐ Yes ☐ No
    - If Yes, reason: ☐ Cost ☐ Distance ☐ No symptoms ☐ Busy ☐ Other ___________

Section E – Economic Impact

1. **In the past 12 months, how many times have you been referred to another health facility for your conditions?**
   ☐ 0 ☐ 1–2 times ☐ 3–4 times ☐ 5 or more

2. **For your most recent referral, please indicate:**

- Facility referred to: ____________________________
- Distance from your home: _____ km
- Travel time (one way): _____ hours/minutes

3. **Direct costs you incurred for the most recent referral visit (approximate amounts in ₹):**
   a) Transport to and from the referred facility: ₹_____
   b) Food and refreshments during travel/stay: ₹_____
   c) Lodging (if overnight stay was required): ₹_____
   d) Any fees paid at the referred facility: ₹_____
   e) Cost of medicines purchased due to referral: ₹_____
   f) Cost of diagnostic tests done at referred facility: ₹_____

4. **Indirect costs for the most recent referral:**
   a) Number of workdays you lost (patient): _____ days
   b) Number of workdays lost by accompanying family member/caregiver: _____ days
   c) Estimated income loss (total): ₹_____

5. **Did you need to arrange additional child care or dependent care during your referral visit?**
   ☐ Yes (cost: ₹_____) ☐ No ☐ Not Applicable

6. **How were the costs for the referral covered?**
   ☐ Out-of-pocket payment
   ☐ Borrowed money/loan
   ☐ Sold assets
   ☐ Covered by insurance
   ☐ Government scheme (specify): ___________________
   ☐ Other: ___________________

7. **In the past 12 months, have you missed a referral or follow-up appointment due to cost?**
   ☐ Yes ☐ No
   If Yes, what was the main reason?
   ☐ Travel cost too high
   ☐ Could not afford medicines/tests at referred facility
   ☐ Loss of income from missing work
   ☐ Other: ___________________

8. **Have referral and follow-up visits caused your household to spend more than 10% of annual income on healthcare in the last year (catastrophic expenditure)?**
   ☐ Yes ☐ No

9. **If there were no cost barriers, would you have attended all referrals/follow-ups as advised?**
   ☐ Yes ☐ No – Why? ___________________

Section F – Satisfaction & Perceived Quality

1. **Overall satisfaction with referral process** (1–5 scale): ___
2. **Overall satisfaction with follow-up care** (1–5 scale): ___
3. **Main challenge you face in continuing your care**: ___________
4. **Main improvement you suggest for referral and follow-up system**: ___________
