# Supplementary material for: Strengthening continuity of care for multiple long-term conditions in India through integrated referral and follow-up systems (SYNC-RF): A mixed-methods study protocol
Source: PLoS One. 2026 Nov 3;21(11):e0361895. doi: 10.1371/journal.pone.0361895 (PMC13730321; doi:10.1371/journal.pone.0361895)
Supplement: Supplementary file 3 [file pone.0361895.s005.pdf]

Interview Guide – People living with MLTC
(Dash et al)

Instructions for Interviewer

Before starting the interview, please:

1. Review the Participant Information Sheet (PIS) and Consent Form (CIF) with the participant in their preferred language.
2. Ensure informed consent is obtained (signed or thumb impression) before proceeding.
3. Reassure the participant that:
    - Their participation is voluntary.
    - They may skip any question or stop the interview at any time.
    - All responses will be kept confidential and anonymised.
4. Seek permission to audio record the interview for accuracy.
5. Ensure the interview is conducted in a private, comfortable, and interruption-free space.
6. Use open-ended questions, listen actively, and allow pauses for reflection.
7. Use probes to encourage elaboration without leading the participant.

Introduction (for interviewer to read out)

"Thank you for agreeing to take part in this interview. We are speaking to patients with more than one long-term health condition to understand their experiences with referral and follow-up care in the health system. We want to learn about the challenges you face, the things that help, and what could be improved. This will help us identify gaps and develop better ways to coordinate care for patients like you. There are no right or wrong answers – we are interested in your personal experiences. This conversation will take about 20–30 minutes. With your permission, I will audio record it so I can accurately capture your words. Your name or any identifying details will not be used in our reports."

Participant Details

(To be filled in by interviewer before starting main questions)

- **Participant ID:**
- **Date of Interview:**
- **Location:**
- **Interviewer Name:**
- **Age:**
- **Gender:**
- **Marital Status:**
- **Occupation:**
- **Education Level:**
- **Number of Long-Term Conditions:**
- **Main Diagnoses:**
- **Years since first diagnosis:**

Section A – Background and Health Profile

Context setting:

"First, I'd like to understand a little about your health conditions and your usual care routines."

Questions & Probes:

1. Can you tell me about the long-term health conditions you are currently living with?

    - Probe: Which one was diagnosed first? How long ago?
    - Probe: Who diagnosed you? (PHC, private clinic, hospital)
2. How do you usually manage your health conditions on a day-to-day basis?
    - Probe: Medicines, diet, exercise, regular check-ups.
3. Where do you usually go for treatment or follow-up care?
    - Probe: Why do you choose this facility? (distance, trust, affordability)

Section B – Experiences with Referral

Context setting:

"Now I'd like to understand your experiences of being referred from one health facility to another for your care."

Questions & Probes:

1. Can you tell me about the last time you were referred to another facility for your condition?
    - Probe: What was the reason for the referral?
    - Probe: Who made the referral?
2. How was the referral given to you?
    - Probe: Was it written, verbal, or via phone?
    - Probe: Did you receive any documents to take with you?
3. Did you clearly understand why you were referred and what you were expected to do?
    - Probe: Was the purpose of the referral explained in simple language?
4. What happened after you received the referral?
    - Probe: How soon did you go?
    - Probe: What challenges did you face in getting to the referred facility? (transport, cost, distance, time)
5. Did anyone from the original facility follow up to check if you reached the referred place?

Section C – Experiences with Follow-up Care

Context setting:

"Let's now talk about what happened after your referral and how your follow-up care was arranged."

Questions & Probes:

1. After you visited the referred facility, what was the next step in your care?
    - Probe: Did they send you back to your original facility?
2. Were follow-up appointments scheduled for you?
    - Probe: Who decided on the follow-up plan?
    - Probe: Was it clearly explained to you?
3. Have you ever missed a follow-up appointment?
    - Probe: Why? (cost, distance, illness, other priorities)
4. How well do you think the different facilities communicate with each other about your care?
    - Probe: Did you ever have to repeat tests because records were not shared?

Section D – Barriers and Enablers

Context setting:
"I'd like to know more about what made the referral and follow-up process easier or harder for you."
Questions & Probes:

1. What were the main challenges you faced during referral or follow-up?
    - Probe: Travel and transportation
    - Probe: Cost of care or medicines
    - Probe: Loss of income
    - Probe: Waiting times at facilities
    - Probe: Confusing instructions
2. Was there anything that made the process easier for you?
    - Probe: Help from family/friends
    - Probe: Assistance from healthcare workers
    - Probe: Support from NGOs or community health workers

Section E – Economic Impact
Context setting:
"Sometimes, going for referrals or follow-ups can affect a person's finances. I'd like to understand if this happened for you."
Questions & Probes:

1. Did you face any financial difficulties because of the referral or follow-up visits?
    - Probe: Travel costs, medicines, diagnostic tests, accommodation, loss of wages.
2. Did you have to borrow money or use savings to meet these costs?
    - Probe: From whom? Family, friends, moneylender.
3. Have you ever delayed or avoided going for a referral or follow-up because of the expense?
4. In your opinion, what could be done to reduce the financial burden on patients like you?

Section F – Perceptions and Suggestions
Context setting:
"Finally, I'd like to hear your thoughts on how the referral and follow-up system could be improved."
Questions & Probes:

1. Overall, how satisfied are you with the referral process you experienced?
2. How satisfied are you with the follow-up care you receive?
3. What changes or improvements would make the referral process better for patients with multiple long-term conditions?
4. What would help you attend all your follow-up appointments as advised?

Closing the Interview

- Ask: *"Is there anything else you'd like to share about your experiences that we haven't discussed?"*
- Thank the participant for their valuable time and insights.
- Provide contact information for follow-up.

Interview Guide – Caregivers

(Dash et al)

Instructions for Interviewer

Before starting the interview, please:

1. Review the Participant Information Sheet (PIS) and Consent Form (CIF) with the participant in their preferred language.
2. Ensure informed consent is obtained (signed or thumb impression) before proceeding.
3. Reassure the participant that:
    - Their participation is voluntary.
    - They may skip any question or stop the interview at any time.
    - All responses will be kept confidential and anonymised.
4. Seek permission to audio record the interview for accuracy.
5. Ensure the interview is conducted in a private, comfortable, and interruption-free space.
6. Use open-ended questions, listen actively, and allow pauses for reflection.
7. Use probes to encourage elaboration without leading the participant.

Introduction (for interviewer to read out)

"Thank you for agreeing to take part in this interview. We are speaking to caregivers of people with more than one long-term health condition to understand their experiences in helping patients through referral and follow-up care in the health system. We want to learn about the challenges you face, what helps, and what could be improved. This will help us identify gaps and develop better ways to coordinate care for patients. There are no right or wrong answers – we are interested in your personal experiences. The interview will take about 20–30 minutes. With your permission, I will audio record it so that I can capture your words accurately. Your name or any identifying details will not be used in any reports."

Participant Details

(To be filled in by interviewer before starting main questions)

- **Participant ID:**
- **Date of Interview:**
- **Location:**
- **Interviewer Name:**
- **Age:**
- **Gender:**
- **Marital Status:**
- **Occupation:**
- **Education Level:**

- **Relationship to Patient: (e.g., spouse, child, sibling, friend)**
- **Number of Patients Cared For:**
- **Patient's Main Diagnoses:**
- **Years caring for this patient:**

Section A – Background and Caregiving Role

Context setting:

"First, I'd like to understand your role in looking after the patient and the kind of support you provide."

Questions & Probes:

1. Can you tell me about the person you care for and the health conditions they have?
    - Probe: Which condition was diagnosed first?
2. How long have you been involved in their care?
3. What kind of support do you usually provide?
    - Probe: Daily care, help with medicines, hospital visits, emotional support.
4. Do you accompany them to health facilities for consultations or follow-ups?
    - Probe: Always, sometimes, or only for certain visits.

Section B – Experiences with Referral

Context setting:

"Now I'd like to talk about your experiences when the patient was referred to another health facility."

Questions & Probes:

1. Can you recall the last time the patient was referred to another facility?
    - Probe: Reason for referral.
    - Probe: Who made the referral?
2. How was the referral communicated to you and the patient?
    - Probe: Written slip, phone call, verbal advice.
3. Did you understand the reason for the referral and what you needed to do?
4. How soon did you take the patient to the referred facility?
    - Probe: Any delays? Reasons for delays (cost, travel, patient health, personal constraints).
5. Were there any follow-up communications from the original facility after the referral?

Section C – Experiences with Follow-up Care

Context setting:

"Let's talk about what happened after the referral and how the follow-up was arranged."

Questions & Probes:

1. Was a follow-up appointment planned after the referral visit?
    - Probe: Who informed you about it?
2. Were you able to take the patient for all scheduled follow-ups?
    - Probe: Reasons for missing any.

3. Did the health facilities involved share information with each other, or did you have to repeat information or tests?
4. How easy or difficult was it to coordinate follow-up care for the patient?

Section D – Barriers and Enablers

Context setting:

"I'd like to understand what made the process easier or harder for you as a caregiver."

Questions & Probes:

1. What were the main challenges you faced in helping the patient during referrals or follow-ups?
    - Probe: Travel and transport.
    - Probe: Financial costs.
    - Probe: Taking time off work or household duties.
    - Probe: Managing patient's comfort and needs during travel.
2. Was there anything that made the process easier?
    - Probe: Support from healthcare providers, family, community health workers.

Section E – Economic Impact

Context setting:

"Sometimes caring for a patient and taking them for referrals or follow-ups can have a financial impact. I'd like to understand this in your situation."

Questions & Probes:

1. Did you face any extra expenses related to the referral or follow-up visits?
    - Probe: Travel, accommodation, tests, medicines.
2. Did you lose income because you had to take time off work?
3. Did you need to borrow money or use savings?
4. Have you ever delayed or avoided follow-up visits because of the costs?
5. What would make it easier for caregivers like you to manage the costs?

Section F – Perceptions and Suggestions

Context setting:

"Finally, I'd like to hear your thoughts on how referral and follow-up systems could be improved for patients and caregivers."

Questions & Probes:

1. Overall, how satisfied are you with the referral process?
2. How satisfied are you with the follow-up care arrangements?
3. What changes would make it easier for you to support the patient's care?
4. Is there anything else you would like to add about your experience as a caregiver?

Closing the Interview

- Ask: *"Is there anything else you'd like to share that we haven't discussed?"*
- Thank the participant for their time.

Interview Guide – Healthcare Providers
(Dash et al)

Instructions for Interviewer

Before starting the interview, please:

1. Review the Participant Information Sheet (PIS) and Consent Form (CIF) with the participant in their preferred language.
2. Ensure informed consent is obtained (signed or thumb impression) before proceeding.
3. Reassure the participant that:
    - Their participation is voluntary.
    - They may skip any question or stop the interview at any time.
    - All responses will be kept confidential and anonymised.
4. Seek permission to audio record the interview for accuracy.
5. Ensure the interview is conducted in a private, comfortable, and interruption-free space.
6. Use open-ended questions, listen actively, and allow pauses for reflection.
7. Use probes to encourage elaboration without leading the participant.

Introduction (for interviewer to read out)

"Thank you for agreeing to take part in this interview. We are speaking to healthcare providers involved in caring for patients with multiple long-term health conditions to understand how referral and follow-up systems work in practice. We want to hear about your experiences, challenges, and suggestions for improvement. There are no right or wrong answers – we are interested in your professional insights. The interview will take about 20–30 minutes. With your permission, I will audio record the conversation to ensure accurate documentation. Your name or any identifying details will not appear in reports or publications."

Participant Details

(To be filled in by interviewer before starting main questions)

- **Participant ID:**
- **Date of Interview:**
- **Location:**
- **Interviewer Name:**
- **Age:**
- **Gender:**
- **Job Title / Designation:**
- **Facility Type:**
- **Department / Speciality:**
- **Years of Experience in Healthcare:**
- **Years in Current Position:**
- **Number of Patients with MLTCs seen/encountered per month at PHCs:**

Section A – Professional Role and Background

Context setting:

"First, I'd like to know more about your role and experience in caring for patients with long-term health conditions."

Questions & Probes:

1. Can you describe your current role and responsibilities?
2. How long have you been working at this facility?
3. What types of long-term conditions do you most often see in your patients?
4. Do you frequently encounter patients with two or more long-term conditions?

Section B – Experiences with Referral Systems

Context setting:

"Let's discuss your experiences in referring patients with multiple long-term conditions to other facilities."

Questions & Probes:

1. Can you describe the usual process you follow when referring such patients?
    - Probe: Written referral, verbal instructions, electronic referral systems.
2. What are the most common reasons for referrals?
3. How is referral information communicated to the receiving facility?
    - Probe: Official referral forms, phone calls, patient-carried documents.
4. Do you receive feedback or reports from the referred facility?
    - Probe: Timeliness and completeness of feedback.
5. What challenges do you face in making referrals?
    - Probe: Lack of transport for patients, lack of specialists, administrative delays.

Section C – Follow-up Mechanisms

Context setting:

"Now let's talk about follow-up arrangements for these patients after they are referred."

Questions & Probes:

1. How do you ensure follow-up after referral?
    - Probe: Appointment cards, community health worker follow-up, phone calls.
2. How often do patients return for follow-up care?
3. What challenges prevent patients from attending follow-up visits?
    - Probe: Financial, geographical, cultural, or system-level barriers.
4. Do you have a system for tracking patients who miss follow-up appointments?

Section D – Coordination Between Facilities and Programmes

Context setting:

"Coordination between facilities is important for patient care. I'd like to know your experiences in this area."

Questions & Probes:

1. How well do facilities and programmes (e.g., NPCDCS, NPHCE) coordinate for patient care?
2. Are there standard protocols for referral and follow-up in your facility?
3. How is patient information shared across facilities?
    - Probe: Electronic health records, manual transfer, patient hand-carry.
4. What works well in the current coordination system?
5. What needs improvement?

Section E – Barriers and Enablers
Context setting:
"I'd like to know what factors make referral and follow-up systems work well, and what challenges you face."
Questions & Probes:
1. What are the key barriers you face in providing coordinated care for MLTC patients?
    - Probe: Staff shortages, lack of resources, patient compliance, administrative delays.
2. What factors help you in making referrals and ensuring follow-up?
    - Probe: Supportive colleagues, clear protocols, community health worker involvement.

Section F – Economic and Resource Considerations
Context setting:
"Now let's think about the resource and cost-related aspects from the facility's perspective."
Questions & Probes:
1. What resources (staff, equipment, transport) are most needed to improve referral and follow-up systems?
2. Are there any financial constraints that affect your ability to provide timely referrals or follow-up?
3. How does patient affordability affect adherence to your referral advice?

Section G – Suggestions for Improvement
Context setting:
"Finally, I'd like to hear your suggestions for strengthening referral and follow-up systems for patients with MLTCs."
Questions & Probes:
1. What would you change in the current system to make it more effective?
2. Are there successful models or practices you have seen elsewhere that could be applied here?
3. How can communication between facilities be improved?

Closing the Interview
- Ask: *"Is there anything else you'd like to share that we haven't discussed?"*
- Thank the participant for their time.
- Provide contact details for any follow-up.

Interview Guide – Policy and Programme Stakeholders
(Dash et al)

Instructions for Interviewer

Before starting the interview, please:

1. Review the Participant Information Sheet (PIS) and Consent Form (CIF) with the participant in their preferred language.
2. Ensure informed consent is obtained (signed or thumb impression) before proceeding.
3. Reassure the participant that:
    - Their participation is voluntary.
    - They may skip any question or stop the interview at any time.
    - All responses will be kept confidential and anonymised.
4. Seek permission to audio record the interview for accuracy.
5. Ensure the interview is conducted in a private, comfortable, and interruption-free space.
6. Use open-ended questions, listen actively, and allow pauses for reflection.
7. Focus on system-level experiences and perspectives, not individual patient cases.

Introduction (for interviewer to read out)

"Thank you for taking the time to speak with us today. We are interviewing policymakers, programme managers, and health administrators at the state, district, and block levels to understand how referral and follow-up systems for patients with multiple long-term health conditions are organised, implemented, and monitored. We are interested in your perspectives on policies, coordination mechanisms, challenges, and opportunities for improvement. There are no right or wrong answers – your insights will help identify ways to strengthen care coordination and service delivery. The interview will take about 30–40 minutes. With your permission, I will audio record the conversation to ensure accuracy. Your name or identifying details will not appear in any report or publication."

Participant Details

(To be filled in by interviewer before starting main questions)

- **Participant ID:**
- **Date of Interview:**
- **Location:**
- **Interviewer Name:**
- **Gender:**
- **Designation / Job Title:**
- **Organisation / Department:**
- **Level of Operation: (State / District / Block)**
- **Years in Current Position:**
- **Years in Health Sector:**
- **Programmes Overseen: If Applicable**

Section A – Professional Background

Context setting:

"First, I'd like to learn more about your role and responsibilities in relation to healthcare policy, programme implementation, or administration."

Questions & Probes:

1. Can you describe your current role and key responsibilities?
2. How does your work relate to referral and follow-up systems for chronic disease care?
3. Which programmes or initiatives are under your supervision related to non-communicable diseases or long-term care?

Section B – Policies and Guidelines on Referral and Follow-up

Context setting:

"Let's discuss the existing policies and guidelines that influence referral and follow-up systems for patients with multiple long-term conditions."

Questions & Probes:

1. What policies or official guidelines currently exist for referrals and follow-up in your jurisdiction?
    - Probe: NPCDCS, NPHCE, or state-specific protocols.
2. How are these guidelines communicated to facilities and healthcare workers?
3. Are there mechanisms to monitor adherence to these guidelines?
4. What are the strengths and limitations of the current policies?

Section C – Coordination Across Levels of Care

Context setting:

"Coordination between different levels of the health system is important for patient care. We'd like to hear about how this works in your context."

Questions & Probes:

1. How do primary, secondary, and tertiary facilities coordinate referrals for MLTC patients?
2. Are there formal communication channels between facilities?
    - Probe: Electronic systems, written forms, direct calls.
3. How is follow-up information communicated back to the referring facility?
4. What role do programme managers or district/state offices play in supporting this coordination?

Section D – Resources, Infrastructure, and Capacity

Context setting:

"Now let's talk about the resources and capacity needed for effective referral and follow-up systems."

Questions & Probes:

1. What resources are available at different facility levels to support referrals?
    - Probe: Staff, transport, equipment, IT systems.
2. Are there dedicated budgets or funding streams for referral and follow-up activities?
3. What training is provided to healthcare staff on referral processes?
4. Where do you see the biggest resource gaps?

Section E – Monitoring, Data, and Evaluation

Context setting:

"Data collection and monitoring systems are essential for improving referral and follow-up services."

Questions & Probes:

1. What kind of data is collected on referrals and follow-ups?
2. How is this data reported and used for decision-making?
3. Are there standard indicators for measuring performance?
4. How could data systems be strengthened to improve service delivery?

Section F – Barriers and Challenges

Context setting:

"We'd like to discuss the main challenges in implementing effective referral and follow-up systems."

Questions & Probes:

1. What are the main barriers you face in implementing or monitoring referral systems?
    - Probe: Financial, structural, human resource, technological.
2. Are there political or administrative challenges?
3. How do social or cultural factors influence patient compliance with referrals?

Section G – Enablers and Best Practices

Context setting:

"Now let's discuss what's working well."

Questions & Probes:

1. What initiatives or innovations have improved referrals and follow-up in your region?
2. Are there examples of successful models from other states or countries that could be adapted?
3. What role have partnerships or collaborations played?

Section H – Economic and System Impacts

Context setting:

"Let's reflect on the broader economic and system impacts of referral and follow-up mechanisms."

Questions & Probes:

1. How do referral and follow-up systems impact healthcare costs for the system and for patients?
2. Are there ways to make these processes more cost-effective?
3. How do you prioritise resource allocation for long-term condition care?

Section I – Recommendations for Improvement

Context setting:

"Finally, I'd like to hear your suggestions for strengthening these systems."

Questions & Probes:

1. If you could change three things in the current system, what would they be?
2. How can state/district/block health offices better support facilities in referrals and follow-ups?
3. What role should technology play in improving these systems?

Closing the Interview

- Ask: *"Is there anything else you'd like to share that we haven't covered?"*
- Thank the participant for their time.
- Provide contact details for follow-up or withdrawal requests.
