# Supplementary material for: Strengthening continuity of care for multiple long-term conditions in India through integrated referral and follow-up systems (SYNC-RF): A mixed-methods study protocol
Source: PLoS One. 2026 Nov 3;21(11):e0361895. doi: 10.1371/journal.pone.0361895 (PMC13730321; doi:10.1371/journal.pone.0361895)
Supplement: Supplementary file 5 [file pone.0361895.s001.docx]

**S1 Checklist. PRISMA-P 2015 checklist for the systematic review component**

This checklist has been completed for the Phase 1 systematic review component of the wider mixed-methods research programme. Section names are provided rather than page numbers because pagination may change during journal formatting.

| **Section and topic** | **Item No** | **Checklist item** | **Location / response for this protocol** |
| --- | --- | --- | --- |
| **ADMINISTRATIVE INFORMATION** | | | |
| Title: Identification | **1a** | Identify the report as a protocol of a systematic review. | Reported in Methods > Phase 1: Evidence synthesis > Systematic review. The PROSPERO review title is: Strengthening the Chain of Care: A systematic review of referral and follow-up mechanisms for managing multiple long-term conditions. |
| Title: Update | **1b** | If the protocol is for an update of a previous systematic review, identify as such. | Not applicable. |
| Registration | **2** | If registered, provide the name of the registry and registration number. | Reported. Methods > Systematic review states that the review is registered with PROSPERO, Registration ID: CRD420251036215. |
| Authors: Contact | **3a** | Provide name, institutional affiliation, e-mail address of all protocol authors; provide physical mailing address of corresponding author. | Reported in PROSPERO ID CRD420251036215 |
| Authors: Contributions | **3b** | Describe contributions of protocol authors and identify the guarantor of the review. | Reported. The PROSPERO record identifies the first author/corresponding author as review guarantor and contact. |
| Amendments | **4** | If the protocol represents an amendment of a previously completed or published protocol, identify as such and list changes; otherwise, state plan for documenting important protocol amendments. | This protocol is not an amendment |
| Support: Sources | **5a** | Indicate sources of financial or other support for the review. | Reported. Funding section |
| Support: Sponsor | **5b** | Provide name for the review funder and/or sponsor. | Reported. Funding section |
| Support: Role of sponsor or funder | **5c** | Describe roles of funder(s), sponsor(s), and/or institution(s), if any, in developing the protocol. | The funder had no role in developing the protocol, study design, analysis plan, interpretation, or decision to publish. |
| **INTRODUCTION** | | | |
| Rationale | **6** | Describe the rationale for the review in the context of what is already known. | Reported Methods > Phase 1: Evidence synthesis > Systematic review. |
| Objectives | **7** | Provide an explicit statement of the question(s) the review will address with reference to participants, interventions, comparators, and outcomes. | Reported. Abstract Aim and Methods, Methods > Systematic review, and PROSPERO |
| **METHODS** | | | |
| Eligibility criteria | **8** | Specify study characteristics and report characteristics to be used as criteria for eligibility for the review. | Reported. Methods > Systematic review and Table 1 present eligibility criteria |
| Information sources | **9** | Describe all intended information sources with planned dates of coverage. | Reported. Methods > Systematic review lists MEDLINE/PubMed, Embase, CINAHL, Scopus, Web of Science, and Cochrane Library, with trial registries, reference list screening, and forward citation tracking. |
| Search strategy | **10** | Present draft of search strategy for at least one electronic database, including planned limits, such that it could be repeated. | Reported. |
| Study records: Data management | **11a** | Describe mechanisms that will be used to manage records and data throughout the review. | Reported. Methods > Systematic review and PROSPERO |
| Study records: Selection process | **11b** | State the process used for selecting studies through screening, eligibility, and inclusion. | Reported. Methods > Systematic review |
| Study records: Data collection process | **11c** | Describe planned method of extracting data from reports, including piloting forms and duplicate extraction; describe process for obtaining/confirming data from investigators. | Reported. Methods > Systematic review |
| Data items | **12** | List and define all variables for which data will be sought, and any pre-planned data assumptions and simplifications. | Reported. Methods > Systematic review |
| Outcomes and prioritization | **13** | List and define all outcomes for which data will be sought, including prioritization of main and additional outcomes. | Reported. Manuscript Table 1 |
| Risk of bias in individual studies | **14** | Describe anticipated methods for assessing risk of bias of individual studies, including level of assessment and how this will be used in data synthesis. | Reported. Methods > Systematic review |
| Data synthesis | **15a** | Describe criteria under which study data will be quantitatively synthesized. | Reported. Methods > Systematic review. |
| Data synthesis | **15b** | If data are appropriate for quantitative synthesis, describe planned summary measures, methods of handling data, and methods of combining data from studies, including exploration of consistency. | Reported in PROSPERO. |
| Data synthesis | **15c** | Describe any proposed additional analyses, such as sensitivity or subgroup analyses or meta-regression. | Reported in PROSPERO. |
| Data synthesis | **15d** | If quantitative synthesis is not appropriate, describe the type of summary planned. | Reported. Methods > Systematic review |
| Meta-bias(es) | **16** | Specify any planned assessment of meta-bias(es), such as publication bias across studies or selective reporting within studies. | Reported in PROSPERO. |
| Confidence in cumulative evidence | **17** | Describe how the strength of the body of evidence will be assessed, such as GRADE. | Reported in PROSPERO |

Source checklist: PRISMA-P (Preferred Reporting Items for Systematic review and Meta-Analysis Protocols) 2015 checklist. This completed version is intended for PLOS ONE supporting information.
