# Supplementary material for: Strengthening continuity of care for multiple long-term conditions in India through integrated referral and follow-up systems (SYNC-RF): A mixed-methods study protocol
Source: PLoS One. 2026 Nov 3;21(11):e0361895. doi: 10.1371/journal.pone.0361895 (PMC13730321; doi:10.1371/journal.pone.0361895)
Supplement: Supplementary file 6 [file pone.0361895.s002.docx]

Appendix 2: Draft search strategy

**Pubmed**

((((((((((((((((((((Referral Pathway*[Title/Abstract]) OR (Follow-up Pathway*[Title/Abstract])) OR (Follow up Pathway*[Title/Abstract])) OR (referral system*[Title/Abstract])) OR (follow-up system*[Title/Abstract])) OR (follow up system*[Title/Abstract])) OR (Patient Navigation[Title/Abstract])) OR (Integrated Care Model*[Title/Abstract])) OR (referral network*[Title/Abstract])) OR (follow-up network*[Title/Abstract])) OR (follow up network*[Title/Abstract])) OR (referral mechanism*[Title/Abstract])) OR (follow-up mechanism*[Title/Abstract])) OR (follow up mechanism*[Title/Abstract])) OR (referral model*[Title/Abstract])) OR (follow-up model*[Title/Abstract])) OR (follow up model*[Title/Abstract])) OR (continuity of care model*[Title/Abstract])) OR (Care Pathway*[Title/Abstract])) OR (chronic care model*[Title/Abstract])) AND (((((((((((multiple long-term condition*[Title/Abstract]) OR (multimorbid*[Title/Abstract])) OR (multi-morbid*[Title/Abstract])) OR (co-morbid*[Title/Abstract])) OR (comorbid*[Title/Abstract])) OR (chronic disease*[Title/Abstract])) OR (chronic illness[Title/Abstract])) OR (chronic conditions[Title/Abstract])) OR (polymorbid*[Title/Abstract])) OR (long-term conditions[Title/Abstract])) OR (MLTC*[Title/Abstract])) AND ((humans[Filter]) AND (english[Filter]))
